# The importance of disrupting complement activation in acute lung injury

**DOI:** 10.1172/JCI182251

**Published:** 2024-06-17

**Authors:** Rick Kapur, John W. Semple, Alexander P.J. Vlaar

**Affiliations:** 1 Sanquin Research, Department of Experimental Immunohematology, Amsterdam, Netherlands.; 2 Landsteiner Laboratory, Amsterdam University Medical Center, University of Amsterdam, Amsterdam, Netherlands.; 3 Division of Hematology and Transfusion Medicine, Lund University, Lund, Sweden.; 4 Clinical Immunology and Transfusion Medicine, Office of Medical Services, Region Skåne, Lund, Sweden.; 5 Department of Intensive Care Medicine and; 6 Laboratory of Experimental Intensive Care and Anesthesiology, Amsterdam University Medical Center, Amsterdam, Netherlands.

**Keywords:** Immunology, Pulmonology, Complement

**To the editor:** With great interest, we read the article by Cleary et al. (1), which provides valuable insights into the pathogenesis of alloantibody-mediated acute lung injury, such as transfusion-related acute lung injury (TRALI) and rejection of lung transplants. This comprehensive study, using a well-established murine model of antibody-mediated TRALI, reveals the essential requirement of IgG hexamer formation in activating the complement system, leading to the onset of acute lung injury.

The authors suggest that therapeutics aimed at targeting IgG hexamerization may potentially open up doors for therapeutic interventions (1). This is based on several approaches that were applied to block alloantibody-mediated hexamerization in the preclinical mouse model of antibody-mediated TRALI, which resulted in prevention of acute lung injury. The described findings substantially enhance our understanding of antibody-mediated acute lung injury, particularly the role of IgG hexamerization in the initiation of acute lung injury. There are, however, several challenges that should be addressed in the subsequent search for novel and effective therapeutics, particularly considering the translatability of the findings into the real-world clinical settings. In the study by Cleary et al., only prophylactic approaches were evaluated, and therefore their assessments were limited to the time before the emergence of clinical symptoms. In acute and unexpected clinical scenarios, such as TRALI, treatments are greatly needed after symptoms have appeared in order to prevent the high degree of morbidity and mortality. The ability of interventions blocking alloantibody-mediated hexamerization to alter the disease’s trajectory toward acute lung injury, once initiated, remains an essential area for further investigation.

In addition, we believe it would be worthwhile to investigate directly targeting complement activation, a consequence of IgG hexamerization. The critical importance of complement in alloantibody-mediated acute lung injury was highlighted in recent studies, based on anti–major histocompatibility complex I (antibody clone 34-1-2S) (2, 3) and anti-CD36 (4). Furthermore, in a large cohort of 53 patients with TRALI, plasma complement levels were found to be increased (3). Taken together, these studies highlight the importance of antibody Fc-mediated complement activation in the development of acute lung injury. We believe that targeting complement directly, for instance via anti-C5a, may have high potential to inhibit and rescue from alloantibody-mediated acute lung injury (5). Blocking downstream complement will keep the complement membrane attack complex intact, which is essential in critically ill patients to prevent life-threatening secondary infections. This approach deserves further exploration and could also widen the therapeutic window for acute lung injury from antibody-mediated mechanisms to nonantibody alloimmune responses, which have also been shown to be of clinical importance in TRALI and organ transplant rejections. For instance, lung transplant rejections often involve complex immune-mediated mechanisms beyond only the actions of alloantibodies, where complement activation plays an important role. Direct downstream complement inhibitors could, therefore, offer a valuable therapeutic strategy in reducing injury in such contexts, emphasizing the need for broader investigations into complement-targeted therapies.

In summary, the study by Cleary et al. (1) provides insights into the importance of IgG hexamerization in the initiation of alloantibody-mediated acute lung injury and opens up several avenues for further research. The effectiveness of successful therapeutic strategies in an acute setting depends on their ability to halt the course of a disease after symptoms have appeared, which may be achieved by directly targeting the complement system and its secondary effects. Possible nonantibody-mediated mechanisms of complement activation should also be considered. Future studies should tackle these issues to ensure that these innovative treatment approaches can be successfully translated from bench to bedside.

## Version 1 06/17/2024

Electronic publication
